# A Successful Whole Body Therapeutic Hypothermia for Hypoxic Ischemic Encephalopathy During an ECMO Run in a Newborn

**DOI:** 10.3389/fped.2019.00095

**Published:** 2019-03-22

**Authors:** Emel Okulu, Omer Erdeve, Bahar Bingoler Pekcici, Tanil Kendirli, Zeynep Eyileten, Begum Atasay, Saadet Arsan

**Affiliations:** ^1^Division of Neonatology, Department of Pediatrics, Ankara University School of Medicine, Ankara, Turkey; ^2^Division of Developmental Behavioral Pediatrics, Department of Pediatrics, Ankara University School of Medicine, Ankara, Turkey; ^3^Division of Pediatric Intensive Care Unit, Department of Pediatrics, Ankara University School of Medicine, Ankara, Turkey; ^4^Department of Pediatric Cardiovascular Surgery, Ankara University School of Medicine, Ankara, Turkey

**Keywords:** newborn, extracorporeal membrane oxygenation, hypoxic ischemic encephalopathy, therapeutic hypothermia, outcome

## Abstract

Data regarding the safety of using therapeutic hypothermia (TH) with extracorporeal membrane oxygenation (ECMO) in neonates with both hypoxic ischemic encephalopathy (HIE), and respiratory failure are lacking. TH is not associated with an increased incidence of hemostatic complications, but hypothermia may impair coagulation. Herein, we report a case of a newborn who had meconium aspiration syndrome and HIE and underwent both TH and ECMO. He did not have any bleeding or circuit complications, and mortality as short-term outcome along with well-neurodevelopmental outcome.

## Introduction

Therapeutic hypothermia (TH) is the standard treatment for neonates with moderate-to-severe hypoxic ischemic encephalopathy (HIE) that has been shown to improve survival without severe neurodevelopmental disability (1, 2). Although TH is not associated with an increased incidence of hemostatic complications, hypothermia may impair coagulation and accelerate microvascular thrombosis formation (3).

Some infants with HIE may also have respiratory diseases that require extracorporeal membrane oxygenation (ECMO) (4, 5). ECMO has been shown to improve survival and decrease morbidity in infants who met the criteria for this therapy; however, an increased risk of bleeding is observed due to systemic heparinization during ECMO support (6).

Data regarding the safety of using TH with ECMO in neonates with both HIE and respiratory failure are limited. Neonates with HIE who underwent both TH and ECMO are thought to be highly at risk for bleeding (7–10).

Herein, we present a newborn with meconium aspiration syndrome (MAS) and HIE who underwent TH and required ECMO due to respiratory failure.

## Case Report

A 4,200-g-male newborn was born at 41 weeks of gestation from an uneventful pregnancy to a 23-year-old healthy mother via vaginal delivery with meconium-stained amniotic fluid. The infant required resuscitation with intubation in the delivery room with Apgar scores of 1, 3, and 7 at 1, 5, and 10 min, respectively. The cord blood and 30-min arterial blood gas analysis revealed severe metabolic acidosis (pH of 6.77 and 6.92, base deficit of 33 and 20 mmol/L, bicarbonate of 11.8 and 14.2 mmol/L, and lactate of 4.1 and 3.2 mmol/L, respectively). He was treated with surfactant lavage and was placed on conventional mechanical ventilation due to MAS. The whole-body cooling was started at the first hour of his life due to the clinical evidence of HIE as hypotonia, presence of diminished deep tendon reflexes, absence of primitive neonatal reflexes with moderate depression of the neurological activity on amplitude-integrated electroencephalogram (aEEG) monitoring. The Thompson score was 17 and classified as severe encephalopathy according to criteria defined by Thompson et al. (11) and Shankaran et al. (12). He received high-frequency ventilation and nitric oxide due to progressive respiratory insufficiency and pulmonary hypertension (50 mm-Hg) with persistent hypoxemia in the following hours. The infant was referred to our neonatal intensive care unit (NICU) to be evaluated for ECMO support at 10th h of his life. Passive cooling was performed during transport with close monitoring of the body temperature. Upon arrival at the NICU, servo-regulated whole-body therapeutic hypothermia (Tecotherm TecCom, Munich Germany) was continued. Target rectal temperature of 33–34°C was achieved and maintained through the servocontrol throughout the duration of therapy. After 72 h of hypothermia, rewarming was slowly accomplished over 8 h to reach the rectal temperature target of 36°C.

Since no improvement was observed on the respiratory support and the oxygenation index was >35 on last 4 h after admission, venovenous (VV) ECMO was decided at the 14th h after delivery. A bicaval double-lumen cannula (13 Fr, Avalon, Avalon Laboratory, Los Angeles, CA) was placed by the cardiovascular surgeon. ECMO treatment was conducted using a centrifugal pump-driven system (MaquetRotaflow, Maquet Cardiopulmonary AG, Hirrlingen, Germany) and hollow-fiber membrane oxygenators (Maquet Quadrox-iD, Maquet Cardiopulmonary AG, Hirrlingen, Germany).

TH was continued throughout the first 72 h on ECMO according to our cooling protocol. No complications were noted during and after TH, and the aEEG returned to continuous normal voltage. A continuous unfractioned heparin infusion at 10 units/kg/h was administered with the ECMO initiation. The dosage of heparin was adjusted according to the activated clotting time at bedside, which was aimed to keep its level within 160–200 s, and the maximum dose of unfractioned heparin during the ECMO run was 40 units/kg/h. Any short-term bleeding and circuit or patient complications were not observed during the ECMO run. Serial transfontanellar ultrasound imaging revealed no intracranial hemorrhage. The infant was successfully decannulated on the 7th day of the ECMO run. The magnetic resonance imaging was performed on the 10th day of life which was reported as normal. He was discharged with normal neurological findings.

His developmental assessment with Bayley Scales of Infant and Toddler Development III (BSID-III) at 2 years old revealed age-appropriate development in all subscales. His composite scores at cognitive scale was 90 (95% confidence interval [CI]: 83–99, percentile rank: 25%), language scales were 94 (95% CI: 87–102, percentile rank: 34%), and motor scales were 85 (95% CI: 79–94, percentile rank: 16%). His developmental monitoring and support are ongoing.

## Discussion

Although TH is the standard treatment of choice for HIE, information on its use with ECMO has been limited. To the best of our knowledge, this is the first case report of a newborn who underwent TH during the ECMO run due to HIE and respiratory failure with long-term neurodevelopmental outcome in the literature.

TH alone is a relatively safe therapy for HIE when used in referral centers (13, 14). The balanced hemostasis is maintained without any risk for hemorrhage or thrombosis in neonates who are not asphyxiated. Although this evidence is inadequate, asphyxiated neonate seems to have impaired balance in hemostasis (3). ECMO is an invasive life support that requires anticoagulation to suppress hemostatic activation and prevent thrombosis (15). Therefore, theoretical increased risks of bleeding complications existed for TH when combined with ECMO. On the contrary, the increased blood viscosity may also disrupt the membrane oxygenator functions or may cause an embolus (9). However, we did not observe any hemostatic abnormality in our patient.

The benefits of performing TH for the treatment of HIE was first reported in 2005 (16), and followed by the first HIE-diagnosed newborn receiving ECMO for respiratory distress with TH in 2008. The delay of using ECMO with TH shows the concerns about the possibility of additive bleeding complications due to the lack of evidence (17).

MAS is characterized by surfactant dysfunction and lung inflammation. De Luca et al. suggested that TH improves the oxygenation and reduces respiratory requirements via reducing metabolic demand and its beneficial effect on lung inflammation and surfactant activity. Further studies are required to demonstrate the safety and efficacy of cooling in patients with MAS (18, 19).

Newborns receiving ECMO are highly at risk for hypoxic-ischemic brain injury. A series of studies demonstrated that applying mild hypothermia for 24–48 h to infants receiving ECMO is feasible and safe (9, 10). Then, TH during ECMO has been hypothesized to reduce the proportion of infants with brain injury, and the only randomized controlled trial, Neonatal ECMO Study of Temperature, examined infants receiving ECMO for respiratory failure allocated to ECMO with TH vs. ECMO alone. This trial demonstrated that no difference was found in the 2-year neurodevelopmental outcome or short-term complications/outcomes at hospital discharge and recommended to avoid the use of cooling for neuroprotection during ECMO (5). However, this study did not enroll patients with HIE.

Cuevas Guaman et al. showed that the bleeding, complications, or mortality did not differ between newborns with HIE who underwent ECMO alone and those who underwent TH with ECMO. This was a retrospective, observational study that included validated data from ELSO registry and did not have neurodevelopmental follow-up data to access long-term efficacy and safety of TH with ECMO (17). On the other hand, a recent study by Cashen et al. showed that TH during neonatal ECMO is associated with intracranial hemorrhage, and they suggested to evaluate the patient with considering the potential risks before applying TH with ECMO (20).

## Conclusion

Our patient is the only one reported without any bleeding, circuit, patient complications, or mortality as short-term outcome along with well-neurodevelopmental outcomes. We suggest that randomized controlled trials are needed to have a conclusion on long-term outcomes in newborns with HIE who underwent TH with ECMO vs. ECMO alone.

## Ethics Statement

Written informed consent was obtained from the parent of the patient for the publication of this case report.

## Author Contributions

EO and OE gave a substantial contribution in article conception and design. BBP and TK participated in acquisition of data. EO, OE, and BBP drafted the manuscript. ZE, BA, and SA critically revised it. All the authors gave their final approval to this manuscript and agree to be accountable for all aspects of work ensuring integrity and accuracy.

### Conflict of Interest Statement

The authors declare that the research was conducted in the absence of any commercial or financial relationships that could be construed as a potential conflict of interest.
